# Health and Ventilation: Artificial Methods Condemned

**Published:** 1924-03

**Authors:** 


### HEALTH AND VENTILATION.

### ARTIFICIAL METHODS CONDEMNED.

Lecturing at Sheffield in the series of addresses on "Health Education," upon "Health and Ventilation," Dr. H. M. Vernon, Fellow of Magdalen College, Oxford, and Investigator for the Industrial Fatigue Research Board, detailed the results of an elaborate series of observations recently made in the State of New York upon the influence of various systems of ventilation in the public schools. The Commission investigated the health and comfort of over 5,000 children day by day for a period of two or three months, as well as the atmospheric conditions of the class-room in which they were working.

### The Cheaper System the Best.

About two-thirds of the rooms were window ventilated, and some of these rooms were kept, throughout the two or three months of observation, at a temperature of about 59° F., a temperature which most of us would think distinctly too cool, whilst others of them were kept at 66° to 67°, a temperature which many of us would think to be rather too warm. Yet the amount of sickness shown by the children from colds, coughs and bronchitis was nearly the same at the two temperatures. In the fan-ventilated rooms, however, the results were very different, for a distinctly larger proportion (18 per cent.) of the children were absent altogether from bronchitis, tonsilitis, and so on, whilst of those actually present 70 per cent. more were suffering from colds and coughs. Probably this result was due partly to the overheating of the rooms, for they were kept at 68°—69°, instead of 66°—67°, and partly to the character of the ventilation. The fan system caused a rather strong but steady current of warm air to pass across the room, usually from above downwards, whilst the window system caused a weaker but more irregular current of air. There is a good deal of evidence to show that variable air currents are much more healthy and exhilarating than steady air currents. Hence we see that the cheaper and more easily installed system of ventilation was distinctly better than the more expensive and elaborate one.

### Advantages of the Natural System.

In my experience, ventilation in large factories in which numbers of workers are often crowded together is often bad, and arranged on incorrect principles. There are great differences of opinion among ventilating engineers, but the observations I have been making during the last year or so lead me to think that so far as possible a natural system of ventilation ought to be adopted, rather than an artificial one. In a natural system air is admitted through openings in the walls and also beneath the windows, where it should be warmed by radiators. As warm air is lighter than cold air, it ascends to the roof, and passes out through ducts to one or more cowls. Unfortunately this system does always work, and with certain directions of the wind you may find that whilst the warm air passes out through one or two of the cowls, a strong draught of cold air passes in through others, and descends on the heads of the inmates. This difficulty can be overcome, however, by having only one main cowl, and by putting a ventilating fan or hot water pipes in it, in order to assist in the extraction of the air.

### The Stuffiness of the Artificial.

Modern factories are now built with a tremendous area of glass windows and it might be thought that they would be cold in winter; but this is not so if hot-water pipes are placed along the floor below the windows, as they entirely neutralise the cold air currents. In warm weather the hopper windows are opened, and in some factories there are very largt swivel windows as well, which give a splendid currene of fresh air, passing for the most part over the heads of the workers in the factory. In the artificial systems of ventilation it is usual to keep all the windows shut, and by means of powerful fans to drive hot or cold air into the rooms through ducts running along the ceiling. It is often said that these plenum systems of ventilation cause the air of the factory to feel very stuffy and depressing, and this is certainly my own experience.

### Smoke-Free Air.

Supposing that you do have open windows in a factory, it is important that the air admitted should be as free from smoke as possible. If you have a condition of things such as is found in industrial towns with hundreds of huge chimneys discharging smoke, it is manifestly impossible to get pure air. Undoubtedly most of these towns are much more smoky than they need be or ought to be, and a diminution in the smoke would re-act favourably on the health of the inhabitants.

---

### THE ADDITIONS TO GLASGOW VICTORIA INFIRMARY.

The important extension to the Glasgow Victoria Infirmary, which is being undertaken this year, will provide 106 additional beds. During the past twelve months the average daily number of patients has been twenty-eight more than the bed accommodation, so that there has been continued overcrowding. It is twenty years since the last extension was made, and the total cost is expected to be about £100,000, while the beds will require £10,000 a year to maintain. The plan was considered some years ago, but the war led to its postponement, and it is now suggested that the hospital should have a country home to which patients could be moved when the critical stage has been passed. An appeal will be issued, and it is hoped that this will be supported not only in the city but in the outside districts from which so many of the patients are drawn. There is at present a deficit on the ordinary working of the infirmary, and unemployment has caused the weekly contributions to fall by £2,000. In the circumstances, therefore, the extension is conrageous, and we trust that one or two public-spirited citizens will come forward and give the work a good start, since the need for it is undeniable and urgent.

